# Reference ranges of T lymphocyte subsets by single-platform among healthy population in southwest China

**DOI:** 10.1186/s12865-021-00474-0

**Published:** 2021-12-20

**Authors:** Bin Wei, Ying Guo, Liangjun Zhang, Huixiu Zhong, Qiang Miao, Lin Yan, Yangjuan Bai, Weihua Feng, Weiping Liu, Qian Niu, Yi Li

**Affiliations:** 1 Department of Laboratory Medicine/Clinical Research Center of Laboratory Medicine, West China Hospital, Sichuan University, 37 Guo Xue Xiang, Chengdu, 610041 Sichuan Province China; 2 West China Second University Hospital/West China Women’s and Children’s Hospital, Key Laboratory of Birth Defects and Related Diseases of Women and Children, Sichuan University, No. 20, Section 3, Renmin South Road, Wuhou District, Chengdu, 610041 Sichuan Province China; 3 Department of Laboratory Medicine, Zigong First People’s Hospital, Zigong, Sichuan Province China

**Keywords:** Reference ranges, T lymphocyte subsets, Single-platform, Southwest China, Indirect method

## Abstract

**Background:**

Appropriate reference ranges of T lymphocyte subsets are essential for immune status evaluation of patients with immunological diseases. We aim to establish the age- and sex-related reference intervals of T lymphocyte subsets by single-platform for the southwest China population using the indirect method with the data resulting from 53,822 cases of periodic health examination individuals in the Laboratory Information System (LIS) of West China Hospital from 2018 to 2020.

**Methods:**

We used the Box-Cox conversion combined with the Tukey method to normalize the data and eliminate the outliers, and the nonparametric method to estimate the 95% distribution reference intervals.

**Results:**

We initially established the reference ranges of T lymphocyte subsets by single-platform among healthy population in southwest China by indirect method (See text for details). Using the standard normal deviate test (z-test) suggested by Harris and Boyd according to CLSI EP28-A3C, which is more scientific, we found the reference ranges of T lymphocyte subsets should be differentiated by ages and genders since the reference ranges of T lymphocyte subsets by single-platform in different ages and genders are significantly different.

**Conclusions:**

We further demonstrated the absolute count of CD3 + T cell, CD3 + CD4 + T cell, CD3 + CD8 + T cell decreased with aging, which is more marked in men and CD3 + CD8 + T cell count, and the obtained reference intervals were superior to the reference intervals derived from the reagent specification currently in use.

**Supplementary Information:**

The online version contains supplementary material available at 10.1186/s12865-021-00474-0.

## Introduction

T lymphocytes (also called T cells) have many functions, including the establishment and maintenance of immune responses, homeostasis, and memory in human body [1]. Meanwhile, T cells coordinate multiple aspects of adaptive immunity, playing a crucial role in pathogen elimination and tumor surveillance [1–3]. Specific subsets of T cells control this process to keep the immune system in check and prevent autoimmunity. For instance, low CD4 + T cell counts and CD4/CD8 ratio were independent unfavorable prognostic predictors for patients with multiple myeloma, waldenstrom macroglobulinemia and mantle cell lymphoma patients at diagnosis [4–7]. Multiple Sclerosis patients have lower proportion of CD3 + T cells and CD4 + T cells than healthy subjects according to Arneth B's research [8]. To our knowledge, the hall mark of human immunodeficiency virus (HIV) infection is a gradual loss of CD4 + T cells and imbalance in CD4 + T cell homeostasis. The peripheral CD4 + T cells count have served as a biomarker for HIV’s immune suppression and response to treatment [9], predicting tumor infiltration and clinical response [10]. Low CD8 + T cell counts are associated with increased AIDS-related mortality and marked elevations in CD8 + T cell counts after long-term combination antiretroviral therapy are associated with increased non–AIDS-related mortality [11]. Consequently, appropriate reference ranges of T lymphocyte subsets are essential for immune status evaluation of patients with immunological diseases.

Immunophenotyping of peripheral T lymphocyte subsets with monoclonal antibodies via flow cytometry which is a rapid and accurate method for identification of peripheral T lymphocyte subsets has proven to be a useful tool to assess immune status of patients with immunodeficiency, autoimmunity, transplantation, tumor, or infection [10, 12–14]. Nevertheless, there are no universal reference ranges of peripheral T lymphocyte subsets currently on account of distinct regions, ages, genders and ethnic groups, which results in most laboratories using the reference ranges provided by reagent manuals. Meanwhile, the previous T lymphocyte subsets absolute count reference intervals in China were mainly established by dual-platform, which multiplies the flow cytometry-derived T-cell percentage by the absolute lymphocyte count derived from a hematology analyzer to calculate the absolute count. Previous studies reported that using of the bead-based single-platform counting methods significantly reduced inter-laboratory variation in absolute cell counts compared to predicate dual-platform methods. With the increasing application of single-platform for lymphocyte counts in clinical laboratory, the reference intervals for single-platform is needed. According to the CLSI EP28-A3C published by The Clinical and Laboratory Standards Institute, each laboratory should establish its own reference intervals [15]. Few studies have concentrated on the reference ranges of peripheral T lymphocyte subsets, which have been varied across previous studies potentially resulting from differences in age, gender, ethnicity, and environmental factors [16–18]. In China, in spite several studies have focused on different age- and sex-related reference intervals of lymphocyte subsets [19–23], their sample sizes are limited and no researches have been reported in southwest China. Our laboratory currently uses the reference intervals recommended by the reagent manual. Therefore, it is necessary to establish the reference ranges of T lymphocyte subsets by single-platform among healthy population in southwest China.

The indirect method based on the assumption, confirmed by observation, that most results, even on hospital and clinic patients, appear “normal” [15]. It uses mathematical-statistical models to analysis the existing data in the hospital database such as laboratory information systems (LIS) to establish biological reference intervals [15, 24]. In recent years, many scholars have reported researches on reference ranges of laboratory indicators using this method [24, 25]. Nevertheless, it is rare to use this method to analyze the reference intervals of T lymphocyte subsets, especially in southwest China. In this study, we aim to establish the age- and sex-related reference intervals of T lymphocyte subsets by single-platform for the southwest China population using the indirect method with the data resulting from 53,822 cases of periodic health examination individuals in the Laboratory Information System (LIS) of West China Hospital from 2018 to 2020, hoping to provide a reliable judgment criteria for clinical evaluation, diagnosis and treatment.

## Materials and methods

### Subjects

**Fig. 1:**
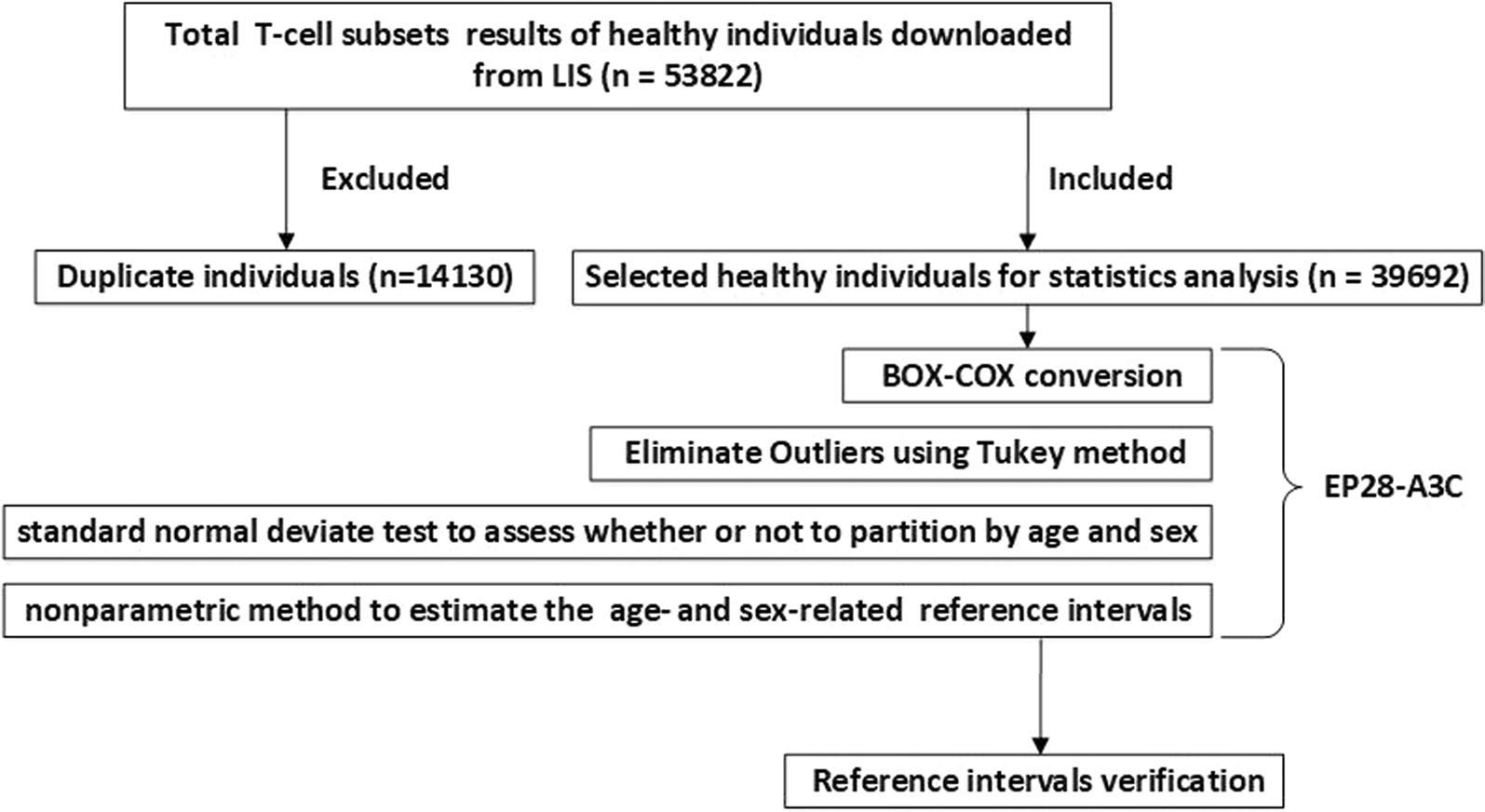
Diagram showing the establishment of reference intervals for T-cell subsets

T lymphocyte subsets test data of 53,822 healthy individuals were collected from Laboratory Information System of West China Hospital of Sichuan University (a university-affiliated hospital with 4300 beds) from 2018 to 2020. To verify whether our results are applicable to Southwest China, we collected T lymphocyte subsets test results of 6134 healthy individuals who examined in Zigong First People's Hospital (a municipal hospital in Sichuan Provence) physical examination center with the same detection algorithm from January to June 2021 for the validation of the obtained reference intervals. Among all the T lymphocyte subsets test results (n = 53,822) in our hospital, we excluded 14,130 duplicate individuals according to physical examination number, name, and age. Eventually, T lymphocyte subsets test data of 39,692 healthy individuals were included. There are 21,087 males (53.13%) and 18,605 females (46.87%) with a median age of 47 years (ranged from 14 to 93 years) (Fig. 1). For the verification population, there are 3482 males (56.77%) and 2652 females (43.23%) with a median age of 48 years (ranged from 17 to 85 years). This study protocol was approved by the Ethics Committee of West China Hospital of Sichuan University and Zigong First People's Hospital, and was performed in accordance with the Declaration of Helsinki. All involved participants provided written informed consent.

### Cell preparation, staining, and flow cytometry

We used FACSCanto II flow cytometer (two lasers, six colors; BD Biosciences, San Jose, CA, USA) with fluorochrome-labeled monoclonal antibodies to confirm the peripheral T lymphocyte subsets phenotypes. Simultaneously, we used four-color monoclonal antibody combinations (LotID:340,499) to determine the circulating lymphocyte subsets. 9 µL of anti-CD45-PerCP/anti-CD3-FITC/anti-CD4-APC/anti-CD8-PE reagent was pipetted into the bottom of a Trucount tube. Subsequently, 50 µL of heparin anticoagulated peripheral blood was distribute into the tube which were soon afterwards capped and vortexed gently. Incubated at room temperature (20–25 °C) for 15 min in the dark, and drop 500 μl of 1X FACS lysing solution into each tube. The tubes were further incubated for 10 min in the dark at room temperature (20–25 °C) and then analyzed using the flow cytometer. For each sample, a minimum of 1000 events were acquired and analyzed by Cell Quest software. Lymphocytes were identified by their strong CD45 expression and low side scatter. The percentages and absolute counts of CD3 + T cells, CD3 + CD4 + T cells, CD3 + CD8 + T cells were automatically calculated using BD Multitest software (BD Biosciences). Internal quality-control procedures were carried out to assess instrument parameters and ensure accurate and reproducible enumeration. Daily calibration of the flow cytometer was performed using Calibrite 3 Beads and APC Beads (BD Calibrite™) for optical laser alignment and optimal hydrodynamic focusing settings, respectively. An external quality control procedure was also completed through participation in a performance assessment conducted by the Clinical Inspection Center of the National Health Commission in China. The original source of cell preparation, staining, and flow cytometry were mainly cited from Niu HQ et al. [21].

### Reference interval establishment methods and verification

The establishment of reference intervals by the indirect method can be roughly summarized into the following 4 steps: (1) Complete sufficient data collection in the hospital database that meets the requirements; (2) Use appropriate methods to transform data with skew distribution; (3) Eliminate outliers or erroneous values in the hospital data; (4) Choose an appropriate method to establish a biological reference interval. First of all, we did the normality test by using the Skewness-Kurtness test. If the skewness value and the kurtosis value are less than 1.96 times the standard deviation, we judged it as a normal distribution. Meanwhile, the Box-Cox transformation was used to transform the non-normally distributed data into approximately normal distribution and Tukey method was used to eliminate outliers. According to the world health organization’s (WHO) age division standard and Chinese age division standard, we divided the individuals into subgroups of gender (male and female) and age (14–30, 31–45, 46–60, 61–75, 76–100 years), and using the standard normal deviate test (z-test) suggested by Harris and Boyd to ascertain whether to divide reference intervals by the subclass. Eventually, we employed the nonparametric method to estimate the 95% distribution reference intervals of T lymphocyte subsets. The documents of Clinical and Laboratory Standards Institute (CLSI) EP28-A3C [15] were referred (Fig. 1). Furthermore, we collected T lymphocyte subsets test results of 6134 healthy individuals who examined in Zigong First People's Hospital (a municipal hospital in Sichuan Provence) physical examination center with the same detection algorithm and platform from January to June 2021 for validation. We explored the ratio of verified individuals outside the reference intervals we set up. When the rate was less than 5%, we consider that the reference interval was verified [15].

### Statistical analysis

The data were analyzed by SPSS version 19.0 for Windows (SPSS Inc., Chicago, IL, USA). Continuous data were checked for normal distribution by the Skewness-Kurtness test. The z-test were used to determine whether to divide the reference intervals according to the subclass. Using Stata 15.0 statistical software performs Box-Cox normality transformation of data (undetermined parameters λ are obtained by maximum likelihood method). The Kruskal–Wallis test and Mann–Whitney U test were used to compare the T lymphocyte subsets results of differences age and sex. GraphPad Prism 7 was used to make charts. A value of *P* < 0.05 was considered statistically significant.

## Results

### Distribution of data and elimination of outliers

T-lymphocyte subsets test results are non-normally distributed by the Skewness-Kurtness test. After BOX-COX conversion, they all show an approximately normal distribution (Table 1). We used the Tukey method to eliminate 126, 352, 417, 506, 550, 444 cases of outliers and finally obtained 39,566, 39,340, 39,275, 39,186, 39,142, 39,248 reference individuals for CD3 + T c3ell (%), CD3 + CD4 + T cell (%), CD3 + CD8 + T cell (%), CD3 + T cell count (cells/ul), CD3 + CD4 + T cell count (cells/ul), CD3 + CD8 + T cell count (cells/ul), respectively (Table 2).

**Table 1:** The skewness and kurtosis of T- lymphocyte subsets before and after BOX-COX conversion

	Before BOX-COX conversion		After BOX-COX conversion	
	Skewness	Kurtosis	Skewness	Kurtosis
CD3 + T cell (%)	− 0.668	0.821	− 0.057	− 0.208
CD3 + CD4 + T cell (%)	0.115	0.094	− 0.001	0.114
CD3 + CD8 + T cell (%)	2.899	104.937	− 0.007	1.234
CD3 + T cell count (cells/ul)	1.084	2.751	− 0.003	0.411
CD3 + CD4 + T cell count (cells/ul)	1.254	3.496	− 0.008	0.538
CD3 + CD8 + T cell count (cells/ul)	1.665	7.092	− 0.021	0.322

**Table 2:** λ value for BOX-COX conversion and number of eliminated outliers use Tukey method in T-cell subsets

	λ	N of outliers	N of individuals eventually included
CD3 + T cell (%)	2.450906	126	39,566
CD3 + CD4 + T cell (%)	0.828792	352	39,340
CD3 + CD8 + T cell (%)	0.309667	417	39,275
CD3 + T cell count (cells/ul)	0.1329571	506	39,186
CD3 + CD4 + T cell count (cells/ul)	0.1203612	550	39,142
CD3 + CD8 + T cell count (cells/ul)	0.0676566	444	39,248

### Reference ranges of T lymphocyte subsets

**Fig. 2:**
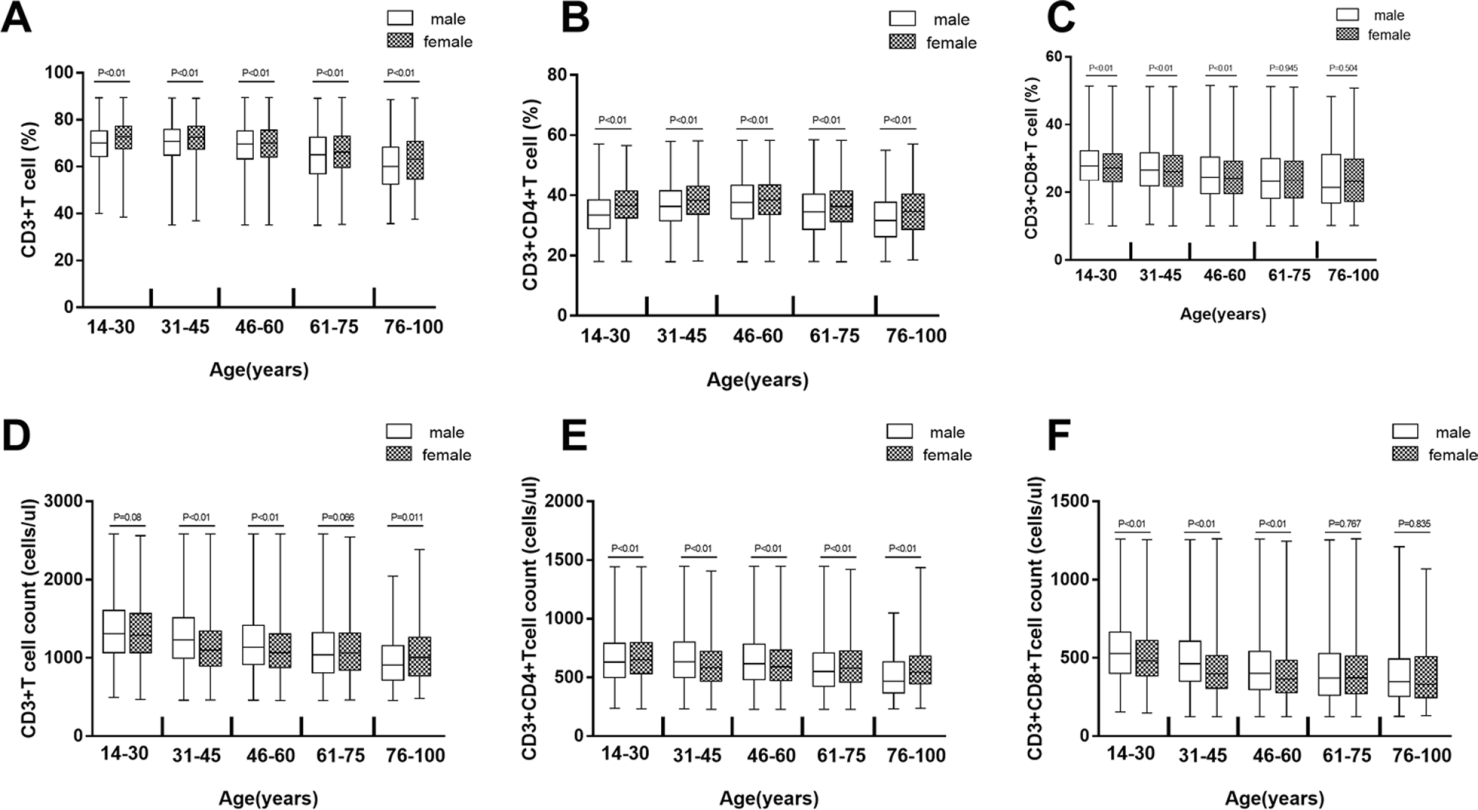
Sex-related **a** CD3 + T cell (%), **b** CD3 + CD4 + T cell (%), **c** CD3 + CD8 + T cell (%), **d** CD3 + T cell count (cells/ul), **e** CD3 + CD4 + T cell count (cells/ul), **f** CD3 + CD8 + T cell count (cells/ul) among different age groups. The tops and bottoms of the boxes represent the minimum to maximum, with the height of the box represents the interquartile range, covering 50% of the values. The line through the middle of each box represents the median

We grouped the data by gender and age. Z-test results showed that it is necessary to set gender- and age-specific reference ranges by subclass. The levels of T lymphocyte subsets were significantly different among age and gender groups (*P* < 0.01, Table 3). Sex-related T lymphocyte subsets among the different age groups are shown in Fig. 2. The reference range, median and individual number of T lymphocyte subsets in different genders and age groups are shown in Tables 3 and 4 respectively.

**Table 3:** Reference ranges of T lymphocyte subsets in different genders and age groups

	Male (2.5th–97.5th)						Female (2.5th–97.5th)						*P* value*
	14–30	31–45	46–60	61–75	76–100	*P* value	14–30	31–45	46–60	61–75	76–100	*P* value	
CD3 + T cell (%)	51.72–83.08	51.60–83.80	48.70–84.00	41.54–83.20	36.28–83.80	< 0.01	56.00–84.20	54.40–84.50	51.50–83.50	46.40–83.30	40.27–84.74	< 0.01	< 0.01
CD3 + CD4 + T cell (%)	21.70–47.90	23.10–51.50	22.90–53.30	20.30–52.20	19.11–50.80	< 0.01	24.70–49.70	25.50–51.60	24.90–52.90	22.30–51.50	21.20–51.99	< 0.01	< 0.01
CD3 + CD8 + T cell (%)	16.40–42.60	14.60–43.33	12.60–43.42	11.80–45.00	10.90–46.81	< 0.01	16.30–41.10	15.10–41.30	13.10–41.50	11.82–43.03	11.24–45.02	< 0.01	< 0.01
CD3 + T cell count (cells/ul)	714–2300	651–2177	600–2103	525–1997	493–1753	< 0.01	688–2155	602–1964	574–1934	556–1974	542–1941	< 0.01	< 0.01
CD3 + CD4 + T cell count (cells/ul)	328–1225	322–1205	305–1191	272–1113	249–923	< 0.01	347–1161	301–1089	298–1106	277–1112	266–1074	< 0.01	< 0.01
CD3 + CD8 + T cell count (cells/ul)	251–1037	204–982	169–939	153–964	138–1026	< 0.01	225–960	185–825	164–832	157–906	149–948	< 0.01	< 0.01

*male VS female use Mann–Whitney U test

**Table 4:** Median and individual number of T lymphocyte subsets in different genders and age groups

	Male						Female					
	14–30	31–45	46–60	61–75	76–100	Total	14–30	31–45	46–60	61–75	76–100	Total
CD3 + T cell (%) (N)	70.05 (1926)	70.80 (7178)	69.70 (9081)	65.10 (2574)	60.10 (249)	69.70 (21,008)	72.80 (2321)	72.60 (6450)	70.00 (7280)	66.20 (2326)	63.20 (181)	71.00 (18,558)
CD3 + CD4 + T cell (%) (N)	33.50 (1921)	36.30 (7147)	37.60 (9015)	34.50 (2537)	31.60 (240)	36.40 (20,860)	36.60 (2316)	38.30 (6434)	38.50 (7234)	36.30 (2314)	34.60 (182)	37.90 (18,480)
CD3 + CD8 + T cell (%) (N)	27.80 (1923)	26.50 (7146)	24.40 (8992)	23.30 (2515)	21.40 (237)	25.40 (20,813)	27.10 (2321)	26.00 (6439)	24.00 (7244)	23.50 (2288)	23.20 (170)	25.20 (18,462)
CD3 + T cell count (cells/ul) (N)	1309 (1906)·	1229 (7100)	1137 (8989)	1038 (2526)	910 (240)	1169 (20,761)	1289 (2304)	1098 (6411)	1066 (7234)	1063 (2296)	1006 (180)	1104 (18,425)
CD3 + CD4 + T cell count (cells/ul) (N)	630 (1905)	633 (7089)	618 (8958)	552 (2536)	469 (242)	614 (20,730)	651 (2295)	582 (6395)	590 (7232)	581 (2309)	544 (181)	593 (18,412)
CD3 + CD8 + T cell count (cells/ul) (N)	528 (1910)	463 (7129)	400 (8999)	372 (2520)	348 (234)	430 (20,792)	482 (2315)	398 (6427)	365 (7237)	373 (2301)	331 (176)	392 (18,456)

### Correlation between T lymphocyte subsets and age in different sex groups

**Fig. 3:**
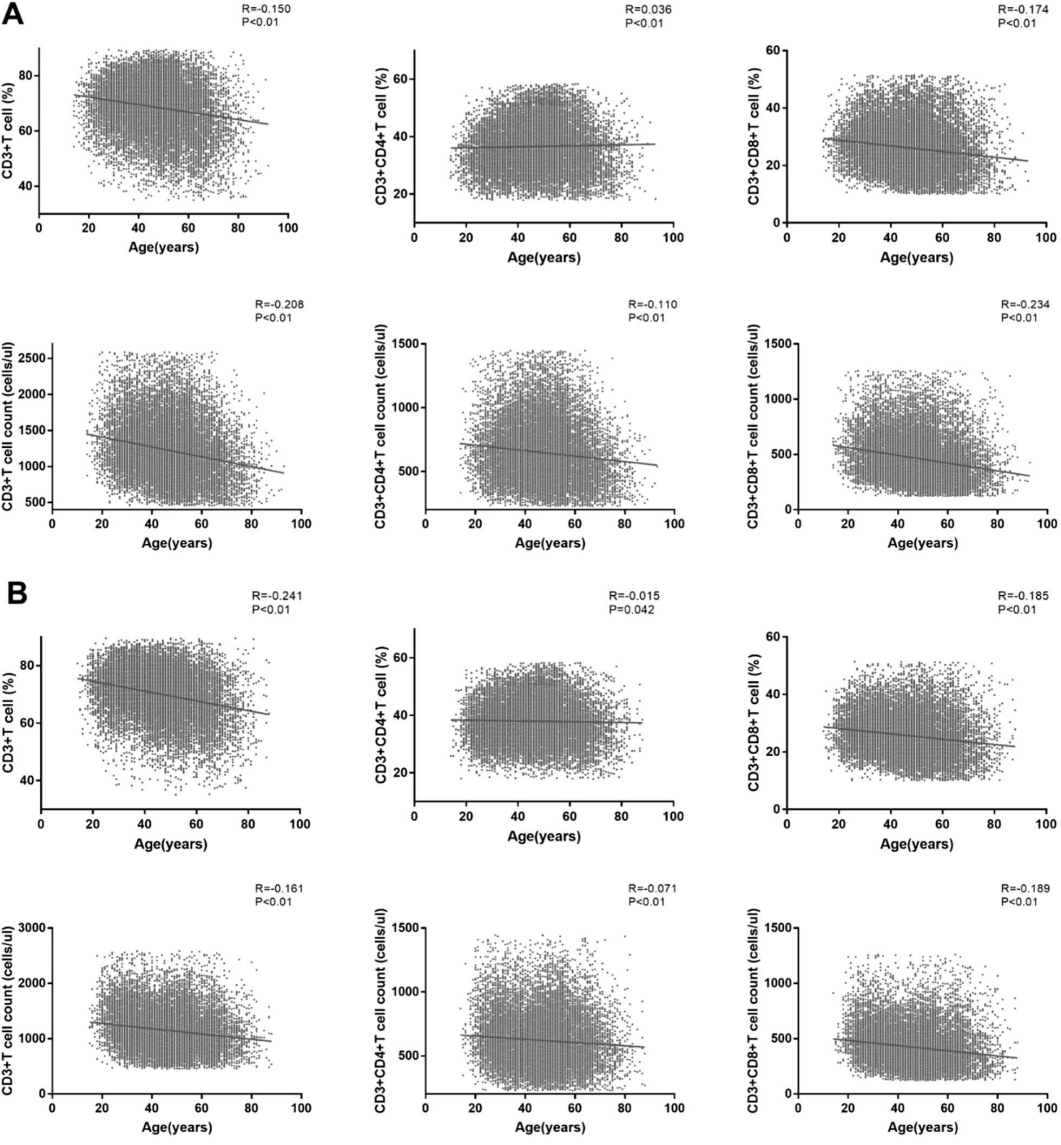
Correlation between T lymphocyte subsets and age in different sex groups. **a** Male. **b** Female

The correlation coefficients between CD3 + T cell (%), CD3 + CD4 + T cell (%), CD3 + CD8 + T cell (%), CD3 + T cell count (cells/ul), CD3 + CD4 + T cell count (cells/ul), CD3 + CD8 + T cell count (cells/ul) and age were − 0.150, 0.036, − 0.174, − 0.208, − 0.110, − 0.234 in male and − 0.241, − 0.015, − 0.185, − 0.161, − 0.071, − 0.189 in female (Fig. 3). The slopes for CD3 + T cell (%), CD3 + CD4 + T cell (%), CD3 + CD8 + T cell (%), CD3 + T cell count (cells/ul), CD3 + CD4 + T cell count (cells/ul), CD3 + CD8 + T cell count (cells/ul) were − 0.14, 0.01, − 0.10, − 6.85, − 2.15, − 3.51 in male and − 0.17, − 0.01, − 0.09, − 4.75, − 1.27, − 2.34 in female (Additional file 1: Table S2), which are more obvious in male and absolute counting.

### Verification and comparison of reference intervals

**Fig. 4:**
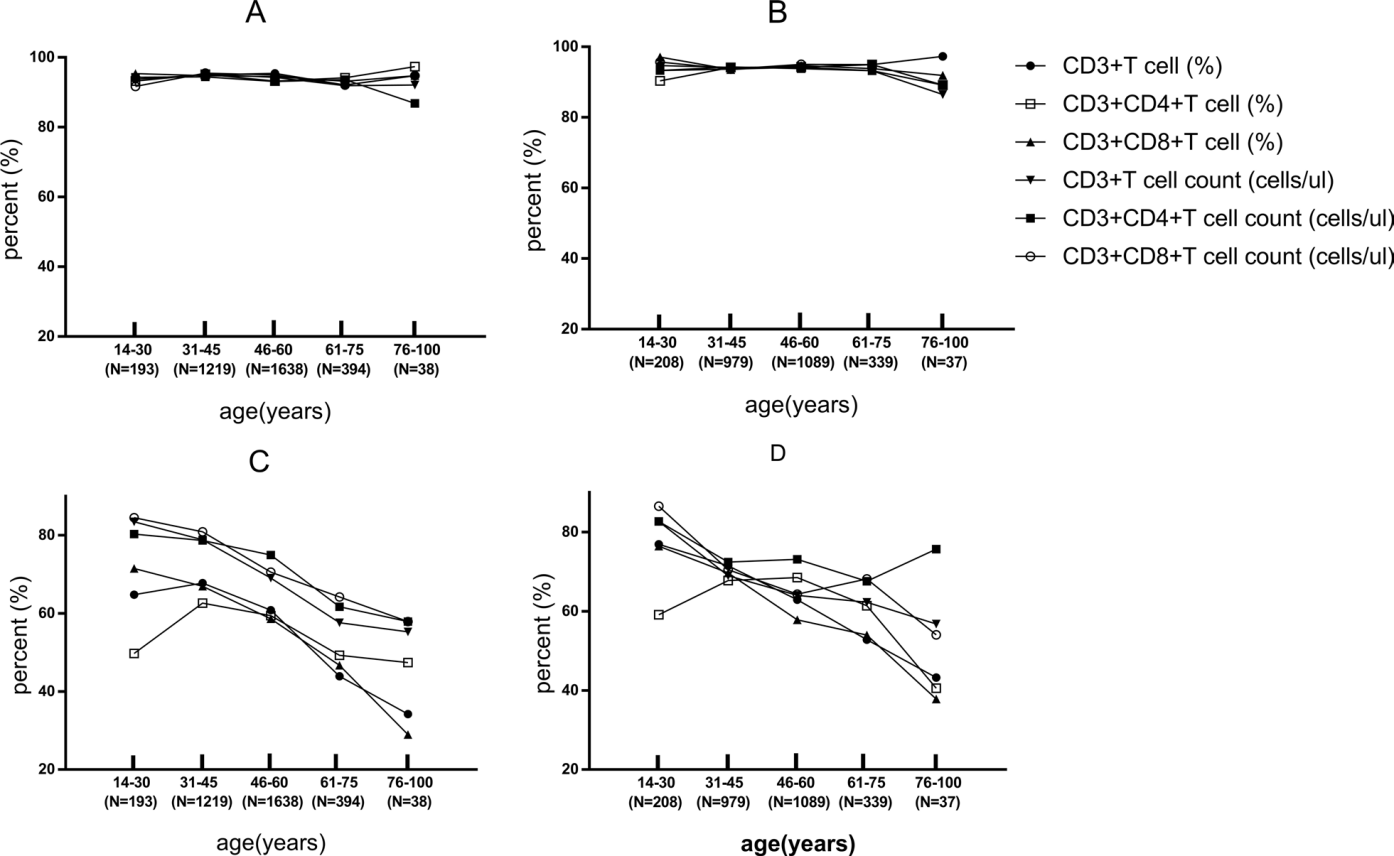
The verifying of T lymphocyte subsets reference intervals by indirect method (**a**: male and **b**: female) and the reagent specification (**c**: male and **d** female). The percent (%) means the ratios of verification individuals inside the reference intervals of different age groups and genders

According to the reagent specification, the T lymphocyte subsets reference intervals currently in use were 66.90–83.10, 33.19–47.85, 20.40–34.70, 941–2226, 471–1220, 303–1003 for CD3 + T cell (%), CD3 + CD4 + T cell (%), CD3 + CD8 + T cell (%), CD3 + T cell count (cells/ul), CD3 + CD4 + T cell count (cells/ul), CD3 + CD8 + T cell count (cells/ul) respectively, regardless of the age and sex. Although the ratios of verification individuals outside the reference intervals by indirect method were partly less than 5% account for the limited sample size in different gender and age groups (Fig. 4a and b), compared with the reference intervals derived from the reagent specification currently in use, it has been greatly improved (Fig. 4c and d). There was a statistically significant difference in the ratio of positive judgments between different reference intervals in the verification population (*P* < 0.01).

## Discussion

Appropriate and reliable reference ranges of T lymphocyte subsets adapted to local population are essential for the immune status evaluation of patients with immunological diseases. In the present study, the single-platform method was used to calculate the absolute number of lymphocyte subgroups (Quantitative microspheres detect the number of lymphoid subsets). This method can directly measure the number of cells, and the result is more objective and accurate, without the error caused by different methods of instrument. We initially evaluated the sex- and age-related reference ranges of CD3 + T cell (%), CD3 + CD4 + T cell (%), CD3 + CD8 + T cell (%), CD3 + T cell count (cells/ul), CD3 + CD4 + T cell count (cells/ul), CD3 + CD8 + T cell count (cells/ul) using flow cytometry among healthy population in southwest China by indirect method with a large sample size of 39,692 healthy individuals, which facilitated the interpretation of research results and clinical decision-making. Based on our research, we found that the reference ranges of T lymphocyte subsets by single-platform in different ages and genders are significantly different. Additionally, we further demonstrated the absolute count of CD3 + T cell, CD3 + CD4 + T cell, CD3 + CD8 + T cell decreased with aging.

The documents CLSI EP28-A3C recommends the direct method to establish the reference intervals. Nevertheless, the difference in screening criteria and the existence of subclinical status of the disease will directly affect the value of the reference intervals. On account of the direct method is complicated, time-consuming, and expensive, most laboratories directly quote the biological reference intervals recommended by the reagent manufacturer, which may not be suitable for the local population. With the development of information technology, utilizing the large amount of data stored in the LIS to establish a biological reference interval based on mathematical statistics (indirect method) has also been accepted by EP28-A3C and used in many studies [24, 25]. The “indirect” method was proposed 50 years ago [24]. This method is used when it is deemed too difficult to collect samples from healthy subjects (eg, pediatrics). In many studies, data from all hospital patients (or all outpatients) are used to estimate reference intervals, but this method is perhaps more appropriately employed using data from individuals who are relatively healthy [15]. The advantages of the indirect method are mainly in two points: (1) There are sufficient and reliable data sources; (2) The operation of the indirect method is simpler than the direct method, time-saving, and low-cost, which is conducive to the establishment and regular review of reference intervals [15]. In this study, we utilized indirect method to establish the sex- and age-related nonparametric reference ranges of T lymphocyte subsets among healthy population in southwest China. Our results show that the reference ranges of T lymphocyte subsets need to be divided by age and gender. The older the age, the lower the absolute count of T lymphocyte subsets in both male and female, which is in consistent with Tang GX et al.'s research [20]. Niu HQ et al. [21] reported that the 2.5% to 97.5% reference range of percentages (absolute counts, cells/μl) CD3 + CD4 + T cells in 150 healthy volunteers aged 20–70 years was 23.78%–51.07% (360–1127) in Shanxi Province, North China. The 2.5% to 97.5% reference ranges of percentages (absolute counts, cells/μl) CD3 + T cells, CD3 + CD4 + T cells, CD3 + CD8 + T cells in Tang GX et al. 's research [20] and Qin L et al. 's research [19] are shown in Additional file 1: Table S1. Our results are similar with Niu HQ et al. [21] and Tang GX et al. [20] but slightly different from Qin L et al. [19]. The discrepancy may be explained by different sample size, indirect method we used to establish reference value and the variance of ethnic groups. Simultaneously, we found the reference ranges of T lymphocyte subsets should be differentiated by ages and genders, using the standard normal deviate test (z-test) suggested by Harris and Boyd according to CLSI EP28-A3C, which is more scientific.

Meanwhile, our results are quite different from those of people in other regions of the world such as South Florida [26], Qatari [27], Omani [28], Ethiopia [18] and German [29]. It seems that the differences in statistical methods (direct or indirect, nonparametric or parametric), detecting platforms (dual or single), instruments as well as genetic, nutrition and environmental variations between populations in different area might result in the discrepancies in lymphocyte subsets, which further illustrated that region reference ranges for lymphocyte subsets were necessary.

According to our results, compared with male, the reference ranges of CD3 + T cell (%), CD3 + CD4 + T cell (%), CD3 + CD8 + T cell (%), CD3 + CD8 + T cell count were narrower in the whole age groups of female, while the reference ranges of CD3 + T cell count, CD3 + CD4 + T cell count were narrower in ≤ 75 years old groups and wider in the 76–100 age group of female (Additional file 1: Table S3). Meanwhile, in agreement with Qin L et al. [19], higher median values of CD3 + T cell (%), CD3 + CD4 + T cell (%) were observed in the whole age groups of female, while the median values of CD3 + CD8 + T cell count were higher in every age groups of male. The median values of CD3 + CD8 + T cell (%), CD3 + T cell count were higher in ≤ 60 years old groups and lower in > 60 years old groups in male. Nevertheless, the median values of CD3 + T cell (%), CD3 + CD4 + T cell (%), CD3 + CD8 + T cell (%), CD3 + T cell count, CD3 + CD4 + T cell count, CD3 + CD8 + T cell count fall by 9.95%, 1.90%, 6.40%, 30.48%, 25.56%, 34.09% between the extremes of age in male and 9.6%, 2.00%, 3.90%, 21.96%, 16.44%, 31.33% in female (Additional file 1: Table S4), which is more marked in men and absolute counting. According to the absolute values of slopes for T lymphocyte subsets in both male and female, we discovered that the absolute values were higher in male, and compared with relative values of T lymphocyte count, absolute counting dropped even more dramatically. We concluded that the total lymphocyte in the peripheral blood decreases with age relatively in parallel with the absolute counting of T lymphocyte decrease, which resulted in an unobviously decrease in the percentage of T lymphocytes. Contemporaneously, CD3 + CD8 + T cell count decreased most obviously and faster than CD3 + CD4 + T cell count in both male and female. These differences may be explained by different hormonal effects [26] and age-related thymic involution [19], which confirmed the influence of sex and age on peripheral lymphocyte subsets.

Accumulating evidence have suggested sex differences in immune responses [30, 31]. Sex hormones play a major role in shaping T cell responses through controlling gene expression in thymic epithelial cells and regulating innate immune cells [30]. For instance, higher estrogen levels in female drive increased T cell IFN-γ production and predispose female to IFN-γ–mediated autoimmune conditions. Androgen/androgen receptor complexes can directly induce anti-inflammatory IL-10 expression by CD4 + T cells, which has been proposed to underlie male protection from central nervous system (CNS) autoimmunity [30]. The broad spectrum of changes affecting the immune system in old age are known as immunosenescence. One of the major causes of immunosenescence of T cells is the involution of the thymus [32, 33]. The rate of thymic T-cell output (around 2 × 10^6^ cells per day at the peak) declines over time, with an estimated half-life of about 16 years in humans [34]. The frequency of naïve T cells is reduced in the periphery and in lymphoid organs in old age, particularly within the CD8 + T cell compartment. The composition of the CD4 + T cell compartment is more stable during aging and it has been described that homeostatic control mechanisms maintain a relatively large and diverse pool of naïve CD4 + T cells, which only changes substantially in very old age [32]. This may explain CD3 + CD8 + T cell count decreased most obviously and faster than CD3 + CD4 + T cell count between the extremes of age in both male and female. In consequence, the sex- and age-related peripheral T lymphocyte subsets differences may be explained by sex hormones discrepancy and age-related thymic involution.

In our study, the correlation coefficients between CD3 + T cell (%), CD3 + CD4 + T cell (%), CD3 + CD8 + T cell (%), CD3 + T cell count (cells/ul), CD3 + CD4 + T cell count (cells/ul), CD3 + CD8 + T cell count (cells/ul) and age were − 0.150, 0.036, − 0.174, − 0.208, − 0.110, − 0.234 in male and − 0.241, − 0.015, − 0.185, − 0.161, − 0.071, − 0.189 in female. According to Niu HQ et al. [21], the correlation coefficients between CD3 + CD4 + T cell (%) and age is 0.294. Tang GX et al. [20] reported the correlation coefficients between CD3 + T cell (%), CD3 + CD4 + T cell (%), CD3 + CD8 + T cell (%), CD3 + T cell count (cells/ul), CD3 + CD4 + T cell count (cells/ul), CD3 + CD8 + T cell count (cells/ul) and age were − 0.318, 0.129, − 0.391, − 0.656, − 0.555, − 0.624 without regard to gender. Qin L et al. [19] found that the correlation coefficients between CD3 + T cell count (cells/ul), CD3 + CD4 + T cell count (cells/ul), CD3 + CD8 + T cell count (cells/ul) and age were − 0.103, − 0.175, − 0.154. Our results are similar with Niu HQ et al. [21] and Qin L et al. [19] but slightly different from Tang GX et al. [20]. The percent of CD3 + T cell, CD3 + CD8 + T cell and the numbers of CD3 + T cell count (cells/ul), CD3 + CD4 + T cell count (cells/ul), CD3 + CD8 + T cell count (cells/ul) were all weak negatively correlated with age in both male and female (*P* < 0.01), while the percent of CD3 + CD4 + T cell was weak positively correlated with age in male but weak negatively in female (*P* < 0.01). The reason needs to be further studied.

The ratios of verification individuals outside the reference intervals derived from the reagent specification currently in use according to the reagent specification were 13.46–71.05%, which will cause overdiagnosis and excessive intervention, causing unnecessary psychological burden and economic burden on the individuals. Moreover, as the age increases, the ratios in the reference interval decreases in both male and female, which further illustrates we should divide the reference interval by age. The ratios of verification individuals outside the reference intervals established by indirect method were 2.88–13.51%. it has been greatly improved and was superior to the reference intervals currently in use.

This study has several limitations. Firstly, the indirect method used in this study has specific restrictions for the inclusion and exclusion of reference individuals and may include some non-healthy individuals. Secondly, due to the limited experimental conditions, we cannot do more analysis of lymphocyte subpopulations such as B and NK lymphocyte. Finally, according to Tang GX et al. [20] and Qin L et al. [19], the increase of function in CD4 + and CD8 + T cells with increasing age is to maintain certain degree of immune function, as the numbers of CD4 + and CD8 + T cells have been declining during life. Moreover, Niu HQ et al. [21] and Togashi Y et al. [35] reported that the abundance of Treg cells in the peripheral blood tends to increase with aging. Botafogo V et al. [36] and Garcia-Prat M et al. [37] expounded that naïve CD4 + T cells tended to decrease with age, in parallel with the reduction in thymic function. Nevertheless, we have not tested the CD4 + T cell subpopulations and the function of CD4 + and CD8 + T cells, which is warranted in our further studies.

In conclusion, we initially established the reference ranges of T lymphocyte subsets by single-platform among healthy population in southwest China by indirect method. We found that the reference ranges of T lymphocyte subsets by single-platform should be differentiated by ages and genders. Additionally, we further demonstrated the absolute count of CD3 + T cell, CD3 + CD4 + T cell, CD3 + CD8 + T cell decreased with aging. The age-related decreasing is more marked in men and CD3 + CD8 + T cell count.

## Supplementary Information

**Additional file 1.** Supplement Table 1–Table 4.

## Data Availability

The datasets generated and/or analysed during the current study are not publicly available due our hospital doesn't allow us to upload raw data on large numbers of patients (we can provide the results of the statistical process if necessary) but are available from the corresponding author on reasonable request.

## Abbreviations

LIS: Laboratory information system
HIV: Human immunodeficiency virus
IQR: Interquartile range
WHO: World health organization
CLSI: Clinical & laboratory standards institute
IFN-γ: Interferon γ
IL-10: Interleukin 10
