# Supplementary material for: Reference ranges of T lymphocyte subsets by single-platform among healthy population in southwest China
Source: BMC Immunol. 2021 Dec 20;22:80. doi: 10.1186/s12865-021-00474-0 (PMC8690880; doi:10.1186/s12865-021-00474-0)
Supplement: Supplementary file 1 — Additional file 1. Supplement Table 1–Table 4. [file 12865_2021_474_MOESM1_ESM.docx]

Supplement Table 1 The 2.5% to 97.5% reference ranges of percentages (absolute counts, cells/μl) CD3+T cells, CD3+CD4+T cells, CD3+CD8+T cells in Tang GX et al. ^[20]^ and Qin L et al. ^[19]^

| Tang GX et al. ^[20]^ | **Children**  **(1-5 years)** | **adolescents (5-18 years)** | **Adults**  **(18-65 years)** | **Elders**  **(>65 years)** |
| --- | --- | --- | --- | --- |
| CD3+T cells% (cells/μl) | 56.62-78.24 (1214-3832) | 57.84-80.08 (1077-3035) | 51.55-81.59 (681-2021) | 44.50-76.68 (589-1712) |
| CD3+CD4+T cells% (cells/μl) | 25.56-45.85 (635-1979) | 23.59-49.08 (560-1653) | 24.82-51.33  (360-1074) | 22.27-49.13  (367-1007) |
| CD3+CD8+T cells% (cells/μl) | 16.69-33.93 (426-1553) | 18.31-38.41 (397-1382) | 13.88-38.73 (180-847) | 8.16-35.13 (116-681) |
| Qin L et al. ^[19]^ | **Young**  **(19-44 years)** | **middle-aged (45-64 years)** | **elder**  **(45-64 years)** |  |
| CD3+T cells% (cells/μl) | 66.05-67.19 (1375-1431) | 65.55-67.80 (1312-1424) | 57.91-65.56 (1071-1323) |  |
| CD3+CD4+T cells% (cells/μl) | 32.43-33.40 (674-706) | 33.91-35.87 (677-738) | 32.72-38.90 (610-787) |  |
| CD3+CD8+T cells% (cells/μl) | 28.01-29.03 (589-621) | 25.96-27.99 (528-595) | 19.96-26.21 (374-522) |  |

Supplement Table 2 The slopes between T lymphocyte subsets and age in different sex groups

|  | Male | Female |
| --- | --- | --- |
| CD3+T cell (%) | -0.14 | -0.17 |
| CD3+CD4+T cell (%) | 0.01 | -0.01 |
| CD3+CD8+T cell (%) | -0.10 | -0.09 |
| CD3+T cell count (cells/ul) | -6.85 | -4.75 |
| CD3+CD4+T cell count (cells/ul) | -2.15 | -1.27 |
| CD3+CD8+T cell count (cells/ul) | -3.51 | -2.34 |

Supplement Table 3 Reference ranges widths of T lymphocyte subsets in different age and sex groups

|  | Male | | | | | Female | | | | |
| --- | --- | --- | --- | --- | --- | --- | --- | --- | --- | --- |
|  | 14-30 | 31-45 | 46-60 | 61-75 | 76-100 | 14-30 | 31-45 | 46-60 | 61-75 | 76-100 |
| CD3+T cell (%) | 31.36 | 32.20 | 35.30 | 41.66 | 47.52 | 28.20 | 30.10 | 32.00 | 36.90 | 44.47 |
| CD3+CD4+T cell (%) | 26.20 | 28.40 | 30.40 | 31.90 | 31.69 | 25.00 | 26.10 | 28.00 | 29.20 | 30.79 |
| CD3+CD8+T cell (%) | 26.20 | 28.73 | 30.82 | 33.20 | 35.91 | 24.80 | 26.20 | 28.40 | 31.21 | 33.78 |
| CD3+T cell count (cells/ul) | 1586 | 1526 | 1503 | 1472 | 1260 | 1467 | 1362 | 1360 | 1418 | 1399 |
| CD3+CD4+T cell count (cells/ul) | 897 | 883 | 886 | 841 | 674 | 814 | 788 | 808 | 835 | 808 |
| CD3+CD8+T cell count (cells/ul) | 786 | 778 | 770 | 811 | 888 | 735 | 640 | 668 | 749 | 799 |

Supplement Table 4 The median values falling ranges of T lymphocyte subsets between the extremes of age

|  | Male | | | Female | | |
| --- | --- | --- | --- | --- | --- | --- |
|  | 14-30 | 76-100 | falling range (%) | 14-30 | 76-100 | falling range (%) |
| CD3+T cell (%) | 70.05 | 60.10 | 9.95 | 72.80 | 63.20 | 9.60 |
| CD3+CD4+T cell (%) | 33.50 | 31.60 | 1.90 | 36.60 | 34.60 | 2.00 |
| CD3+CD8+T cell (%) | 27.80 | 21.40 | 6.40 | 27.10 | 23.20 | 3.90 |
| CD3+T cell count (cells/ul) | 1309 | 910 | 399 (30.48) | 1289 | 1006 | 283 (21.96) |
| CD3+CD4+T cell count (cells/ul) | 630 | 469 | 161 (25.56) | 651 | 544 | 107 (16.44) |
| CD3+CD8+T cell count (cells/ul) | 528 | 348 | 180 (34.09) | 482 | 331 | 151 (31.33) |
